# Nanowell-mediated two-dimensional liquid chromatography enables deep proteome profiling of <1000 mammalian cells†
†Electronic supplementary information (ESI) available. See DOI: 10.1039/c8sc02680g


**DOI:** 10.1039/c8sc02680g

**Published:** 2018-07-18

**Authors:** Maowei Dou, Ying Zhu, Andrey Liyu, Yiran Liang, Jing Chen, Paul D. Piehowski, Kerui Xu, Rui Zhao, Ronald J. Moore, Mark A. Atkinson, Clayton E. Mathews, Wei-Jun Qian, Ryan T. Kelly

**Affiliations:** a Environmental Molecular Sciences Laboratory , Pacific Northwest National Laboratory , Richland , WA 99354 , USA . Email: ryan.kelly@pnnl.gov; b Department of Pathology , Immunology and Laboratory Medicine , University of Florida , Gainesville , FL 32611 , USA; c Biological Sciences Division , Pacific Northwest National Laboratory , Richland , WA 99354 , USA

## Abstract

Multidimensional peptide separations can greatly increase the depth of coverage in proteome profiling. However, a major challenge for multidimensional separations is the requirement of large biological samples, often containing milligram amounts of protein. We have developed nanowell-mediated two-dimensional (2D) reversed-phase nanoflow liquid chromatography (LC) separations for in-depth proteome profiling of low-nanogram samples. Peptides are first separated using high-pH LC and the effluent is concatenated into 4 or 12 nanowells. The contents of each nanowell are reconstituted in LC buffer and collected for subsequent separation and analysis by low-pH nanoLC-MS/MS. The nanowell platform minimizes peptide losses to surfaces in offline 2D LC fractionation, enabling >5800 proteins to be confidently identified from just 50 ng of HeLa digest. Furthermore, in combination with a recently developed nanowell-based sample preparation workflow, we demonstrated deep proteome profiling of >6000 protein groups from small populations of cells, including ∼650 HeLa cells and 10 single human pancreatic islet thin sections (∼1000 cells) from a pre-symptomatic type 1 diabetic donor.

## Abstract 2

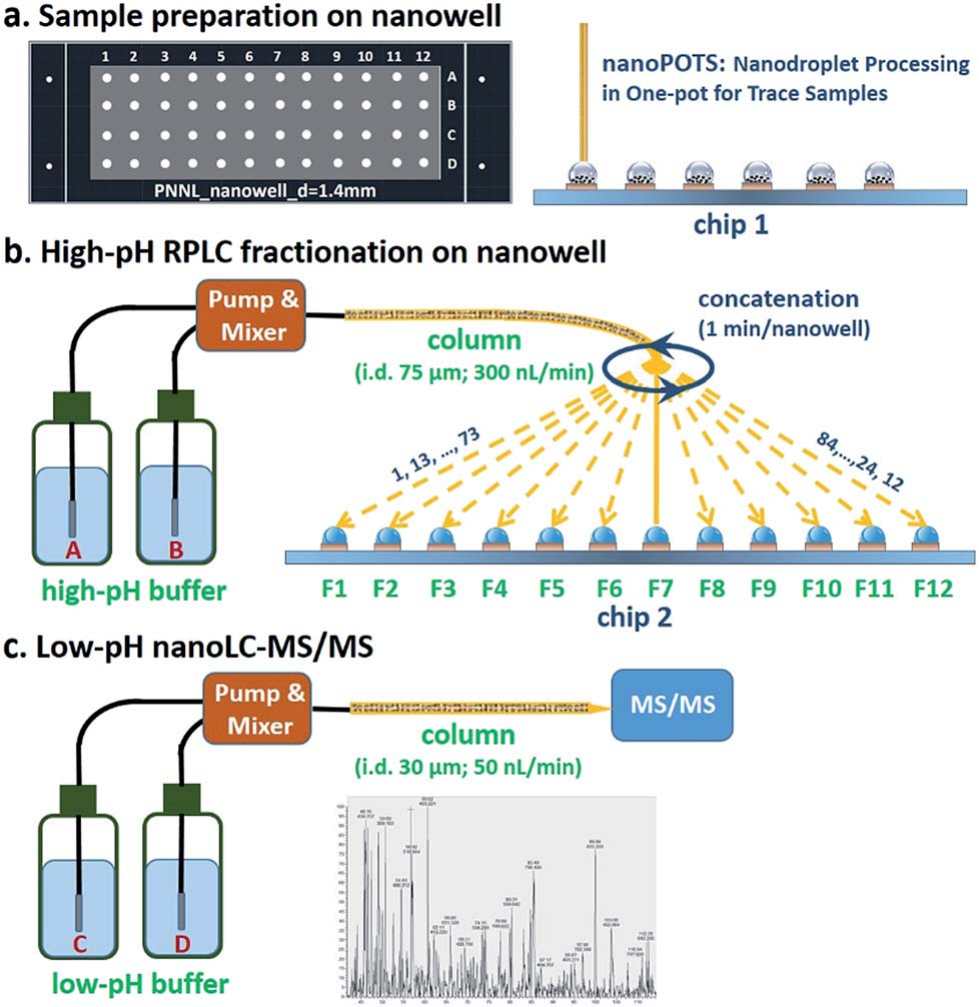
Miniaturized multidimensional peptide separations can greatly increase the coverage in proteome profiling for trace samples.

## Introduction

Global proteomic analyses, in which the entire complement of proteins expressed in a biological system is identified and quantified to the extent practicable, continue to support systems-level inquiry and inform on protein changes in response to environmental perturbations, spatial distribution, developmental state, *etc.*^1^–^4^ In addition to advancing basic research, in-depth global proteomics can enable extensive functional mapping of proteoforms arising from a specific gene, holding great potential for personalized and precision medicine.^5^ However, sensitivity limitations of mass spectrometry (MS)-based proteomics have led to minimum sample requirements of thousands or millions of cells, which precludes the in-depth molecular characterization of limited clinical samples, including fine needle aspiration biopsies, purified primary cells and circulating tumor cells. Increasing sensitivity will thus facilitate minimally invasive sampling and will enhance measurement specificity by resolving the heterogeneity present in biological tissue substructures. Enhancing coverage (the number of identified proteins) adds to the power of proteome measurements, as low-abundance species frequently have great biological significance (*e.g.*, cell signaling) and may otherwise be missed due to insufficient sensitivity of currently available technologies.^6^,^7^ Liquid chromatography (LC) coupled to tandem MS is unrivalled in its power to resolve complex proteomic samples.^8^,^9^ Proteome coverage increases with separation peak capacity, as improved separations simplify the peptide complexity entering the mass spectrometer at a given time, thus reducing spectral complexity and alleviating MS dynamic range limitations, MS/MS undersampling and ionization suppression.^10^,^11^ However, the peak capacity of one-dimensional (1D) reversed-phase (RP) separations only increases incrementally with column length and gradient time.^12^

Multidimensional LC separations can potentially provide far greater resolving power and have generally proven necessary to increase proteome coverage beyond ∼5000 proteins. Implementation of multidimensional separations often involves a first-dimension separation in which the sample is partitioned among a number of discrete fractions, and is based on electrophoresis, strong cation exchange or a high-pH reversed-phase (RP) LC. The second-dimension separation is typically low-pH nanoflow RPLC due to the favourable conditions it provides for efficient and sensitive electrospray ionization-MS. High-pH RPLC is being used increasingly as the first-dimension separation due to rapid chromatographic partitioning and generation of cleaner fractions with low-salt buffers, despite limited orthogonality with the second-dimension nanoLC.^13^ For such pH-modulated two-dimensional (2D) RPLC separations, a relatively high flow first-dimension separation is employed and samples are fractionated into multiwell plates. Solvent is removed using a vacuum-assisted evaporative concentrator (*e.g.*, SpeedVac), and each peptide fraction is reconstituted in tens of microliters of aqueous solution for automated sample handling and injection into the subsequent low-pH RPLC-MS/MS proteomic analysis platform. Unfortunately, the large volumes and the consequent losses of peptides to the surfaces of vials or well plates, transfer tubing and valves have limited 2D LC analyses to applications involving large, often milligram, amounts of starting material. Recently, Mann *et al.*^14^ reported a spider fractionator system to obtain high proteome coverage for samples as small as 0.5 μg. The system was able to identify ∼6000 proteins using 6600 HeLa cells as the starting material. However, the fractions were still collected into vials, where significant surface losses presumably occurred.

Microfluidic systems, with their ability to efficiently manipulate (sub)nanoliter volumes,^15^ are well suited for facilitating multidimensional separations and analyzing trace samples. For example, Ramsey *et al.*^16^ combined micellar electrokinetic chromatography (MEKC) with capillary electrophoresis (CE) to perform highly efficient separations of single-protein tryptic digests. Conversely, Edgar *et al.*^17^ encapsulated CE-separated species into droplets for transfer to a second dimension MEKC separation. Niu *et al.*^18^ utilized a droplet-based microfluidic platform for two-dimensional separations based on LC and CE. However, none of these platforms was utilized for complex samples such as whole proteome tryptic digests or coupled with MS for broad identification. Other droplet microfluidic systems have facilitated label-free detection of trace species by either ESI-MS^19^,^20^ or MALDI-MS,^21^–^23^ and while offering unique advantages for many applications, MS-based proteome profiling continues to rely largely on conventional well plate sample processing and capillary-based separations.

We recently developed a microfluidic nanodroplet-based sample processing platform termed nanoPOTS (Nanodroplet Processing in One pot for Trace Samples) for efficient conversion of biological samples containing low or subnanogram amounts of protein into ready-to-analyze peptides. nanoPOTS uses robotic nanopipetting to dispense nanoliter volumes of reagents into nanowells on a photolithographically patterned glass chip.^24^,^25^ In contrast to many past microfluidic approaches, nanoPOTS avoids surface losses associated with enclosed microchannels or oil-encapsulated droplets. Instead, all processing takes place in an open well format having a total surface area of <1 mm^2^. Evaporation is controlled through the combination of a humidified chamber during dispensing and a contactless cover during extended incubation steps. Using nanoPOTS in combination with ultrasensitive LC-MS/MS, >3000 protein groups have been identified from as few as 10 mammalian cells, which is a depth of proteome coverage not previously achieved for fewer than 1000 cells. nanoPOTS has also been used to identify ∼700 proteins from single mammalian cells.^26^ While nanoPOTS provides an unprecedented depth of proteome coverage at and near the single cell level, it will be challenging to substantially increase proteome coverage without additional breakthroughs in sensitivity due to the use of a 1D nanoLC separation. Here we have adapted the robotic nanopipetting platform and the glass nanowell chips associated with nanoPOTS to enable 2D LC separations of proteome samples as small as 50 ng with an unprecedented depth of proteome coverage. Peptides are separated by high-pH nanoLC, and the eluting peptides are concatenated into glass nanowells prior to being reconstituted in LC-compatible buffer and injected into the second dimension low-pH nanoLC separation. The dramatic reduction in surface-related losses made possible by the nanowell-mediated 2D LC platform enabled profiling of nearly 6000 proteins from just 50 ng of HeLa digest. In combination with nanoPOTS sample processing, populations of ∼650 HeLa cells and <1000 laser microdissected human pancreatic islet cells were profiled with a similar coverage. To the best of our knowledge, there has been no report on in-depth profiling of >5000 proteins using low-nanogram proteomic samples or with <1000 mammalian cells.

## Experimental section

### Chemicals and reagents

Dithiothreitol (DTT) and iodoacetamide (IAA) were products of Thermo Scientific (St. Louis, USA) and were freshly prepared in 50 mM ammonium bicarbonate buffer before use. Trypsin (MS grade) and Lys-C (MS grade) were purchased from Promega (Madison, USA). Unless otherwise noted, all other chemicals and reagents were purchased from Sigma-Aldrich (St. Louis, USA). Deionized water (18.2 MΩ) was produced using a Barnstead Nanopure Infinity system (Los Angeles, USA).

### Fabrication of nanowell chips

Nanowell microfluidic glass chips were fabricated using a photolithographic method (Fig. S1†).^24^ The background of the microfluidic glass chip surrounding the nanowells was rendered hydrophobic with 2% (heptadecafluoro-1,1,2,2-tetrahydrodecyl)dimethylchlorosilane (PFDS) in 2,2,4-trimethylpentane while the nanowells remained hydrophilic. The diameter of the nanowells was 1.4 mm.

### Cell culture

HeLa cells (ATCC, Manassas, USA) were cultured in Eagle's Minimum Essential Medium (EMEM) supplemented with 10% fetal bovine serum (FBS) and 1% penicillin/streptomycin. Cells were incubated at 37 °C in an aerobic environment with 5% CO_2_ and split every 3 days following a standard protocol. Cultured HeLa cells were collected in a 10 mL centrifuge tube and centrifuged at 1200 rpm for 10 min to remove culture media. After washing with PBS buffer three times, the cells were suspended in 1 mL PBS buffer and counted to obtain the concentration.

### Laser microdissection of human pancreatic islets

Ten-micrometer-thick pancreatic tissue slices from pancreata recovered from organ donors through the JDRF Network for Pancreatic Organ Donors with Diabetes (nPOD) program were cut from OCT (optimal cutting temperature compound) blocks using a cryo-microtome and mounted on PEN membrane slides for islet dissection. The slides were briefly fixed with methanol, rinsed with H_2_O to remove OCT, and dehydrated using an alcohol gradient before placing in a desiccator to dry for 8 min. The dehydrated slides were placed on the stage of a laser microdissection microscope (Leica LMD7000). Islets were identified based on autofluorescence and morphology. Dissections were performed under a 10× objective. Laser dissected islets were collected in the cap of a 0.6 mL tube mounted underneath the slides. After dissection, the samples were stored at –80 °C until further analysis. Ethical permission was obtained from the Institutional Review Boards at the University of Florida and Pacific Northwest National Laboratory, and informed consent was obtained from a legal representative of each donor.

### NanoPOTS sample preparation

Cells and reagent solutions were delivered into nanowells using an in-house-developed robotic system,^24^ and a home-built LabView program (Version 2015, National Instruments, Austin, USA) was used to synchronously control the movement of the stages. For cultured cells, after dispensing 100 nL cell suspension into nanowells, accurate cell numbers were obtained by counting under an inverted microscope. For laser microdissected human islets, a high precision tweezer with a tip of 20 μm (TerraUniversal, Buellton, USA) was used to transfer tissue pieces from collection tubes into individual nanowells under a stereomicroscope (SMZ1270, Nikon, Japan).

The nanoPOTS-based sample preparation included the following steps. (1) Cell lysis, protein denaturation, and disulfide reduction: 100 nL DDM (0.2%) solution with 10 mM DTT in 50 mM ammonium bicarbonate was added into each of the nanowells that had been preloaded with cells. Then the nanowell chip was incubated at 70 °C for 30 min. (2) Alkylation of sulfhydryl groups: 100 nL IAA solution (30 mM in 50 mM ammonium bicarbonate) was dispensed into each nanowell, followed by incubating the chip at room temperature in the dark for 30 min. (3) Lys-C digestion: 100 nL enzyme solution containing 0.5 ng Lys-C in 50 mM ammonium bicarbonate was added into the nanowells, followed by incubating the chip at 37 °C for 4 h. (4) Tryptic digestion: 100 nL trypsin solution containing 0.5 ng trypsin in 50 mM ammonium bicarbonate was added into each nanowell, followed by incubating the chip at 37 °C overnight. To minimize liquid evaporation in nanowells, the nanowell chip was placed in a closed chamber maintained at 95% humidity during dispensing procedures, and sealed and placed in a closed humid box during incubation procedures. Digested peptide samples were collected from nanowells and stored in a section of fused silica capillary (150 μm i.d., 360 μm o.d.) and both ends were sealed with Parafilm. The capillary section was stored at –20 °C prior to the first dimension high-pH nanoLC separation.

### High-pH RPLC fractionation

The first dimension LC column (75 μm i.d., 360 μm o.d., 50 cm length) and solid phase extraction (SPE) column (100 μm i.d., 360 μm o.d., 5 cm length) were packed with 3 μm C18 packing material (300 Å pore size, Phenomenex, Terrence, USA) as reported previously.^27^ The capillary section that stored digested peptide samples was connected to the SPE column with a PEEK union (Valco Instruments, Houston, USA). The digested peptide samples were loaded and desalted in the SPE column by infusing 0.1% formic acid in water at a flow rate of 1000 nL min^–1^ for 10 min on a nanoACQUITY UPLC pump system (Waters, Milford, USA). The SPE column was then reconnected to the LC column with a PEEK union. The high-pH RPLC separation was performed at a flow rate of 300 nL min^–1^ on a binary pump system (Thermo Scientific Dionex UltiMate 3000 pump) to deliver a gradient flow to the SPE and LC columns, using 10 mM ammonium formate (pH 8) as mobile phase A and 10 mM ammonium formate in 90% acetonitrile (pH 8) as mobile phase B. Digested peptide samples were eluted from 5% B to 35% B in 65 min, followed by the gradient with a linear increase to 70% B in 15 min and a 10 min wash at 70% B, then a decrease to 2% B in 10 min.

The eluent from the high-pH nanoLC separation was concatenated into nanowells using our recently developed robotic system^24^ with the outlet of the LC capillary column directly mounted on the system in the place of the liquid dispensing tip. The eluent was fractionated into each nanowell at a flow rate of 300 nL min^–1^ for 1 min during the gradient time 12–96 min. For 12 fractions, the eluent was collected into one of the 12 nanowells per minute and then repeated 7 times during the concatenation procedure. For 4 fractions, the eluent was collected into each of the 4 nanowells for 1 min and then repeated 21 times. The fractions were collected using the robotic system and stored in a section of fused silica capillary (150 μm i.d., 360 μm o.d.) using the following procedure. The eluent from the first-dimension separation was allowed to dry in the nanowells and was subsequently reconstituted in 150 nL of 0.1% aqueous formic acid. The reconstituted volume was aspirated into the capillary and washed twice with 200 nL of 0.1% formic acid. The rinse solutions were also aspirated into the capillary as described previously.^24^ The capillary sections with collected fractions were sealed with Parafilm on both ends and stored at –20 °C for the second dimension low-pH nanoLC separation.

### Low-pH nanoLC-MS/MS

Similarly, the second dimension LC column (30 μm i.d., 360 μm o.d., 50 cm length) and SPE column (100 μm i.d., 360 μm o.d., 5 cm length) were packed with 3 μm C18 packing material (300 Å pore size, Phenomenex, Terrence, USA).^27^ The fraction storage capillary was connected to the SPE column with a PEEK union. Then each fraction was loaded and desalted in the SPE column by infusing 0.1% formic acid in water at a flow rate of 1000 nL min^–1^ for 10 min on a nanoACQUITY UPLC pump system (Waters, Milford, USA). Then the SPE column was reconnected to the LC column with a PEEK union. The LC separation flow rate was 50 nL min^–1^, which was split from 300 nL min^–1^ on a binary pump system (Thermo Scientific Dionex UltiMate 3200 pump). A linear 100 min gradient of 5–22% buffer D (0.1% formic acid in acetonitrile) was used for the low-pH nanoLC separation, followed by a gradient of linear increase to 45% in 20 min and a 10 min wash in 90% D, and a final 20 min equilibration with buffer C (0.1% formic acid in water).

Data collection was performed using an Orbitrap Fusion Lumos Tribrid mass spectrometer (ThermoFisher). The temperature of the ion transfer capillary was set to 150 °C to accelerate desolvation and the RF lens was set at a level of 30%. The precursor was scanned at a resolution of 60 000, a scan range of 375–1575, a maximum injection time of 118 ms, and an AGC target of 1 000 000. We used data-dependent acquisition with a fixed cycle time of 3 s for precursor isolation and MS/MS sequencing. Dynamic exclusion was activated to reduce repeat sequencing using an exclusion duration of 60 s and a mass tolerance of 10 ppm. The precursor ions with charge states from 2–7 and minimal intensities over 20 000 were isolated for MS/MS sequencing by high energy collision dissociation (energy level of 30%). The MS/MS scan was performed in the Orbitrap at a resolution of 60 000, a maximum injection time of 118 ms, and an AGC target of 100 000.

### Data analysis

MaxQuant (software version 1.5.3.30)^28^ was used to analyze MS raw data files for database searching and protein/peptide quantification against the UniProtKB/Swiss-Prot human database (Downloaded in 12/29/2016 containing 20 129 reviewed sequences). Trypsin was specified as the protease for enzymatic digestion and two missed cleavages were allowed for each peptide. Carbamidomethylation was set as a fixed modification, and n-terminal protein acetylation and methionine oxidation were set as variable modifications. The minimum peptide length was 6 and the maximum peptide mass was set as 4600 Da. Tandem mass spectra were matched with a tolerance of 5 ppm on the precursor mass and 20 ppm on the fragment mass. Both peptides and proteins were filtered with a maximum false discovery rate (FDR) of 0.01. Label-free quantification (LFQ) was performed in each parameter group containing similar sample sizes. For the correlation of protein intensities between 4-fraction and 12-fraction analyses, Match Between Runs (MBR) was activated to increase the identification of unsequenced peptides in the 4-fraction sample. Perseus^29^ was used to perform data analysis and extraction.

The MS proteomics data have been deposited to the ProteomeXchange Consortium *via* the PRIDE^30^ partner repository with the dataset identifier PXD010150.

## Results and discussion

### Nanowell-mediated 2D LC platform

**Fig. 1:**
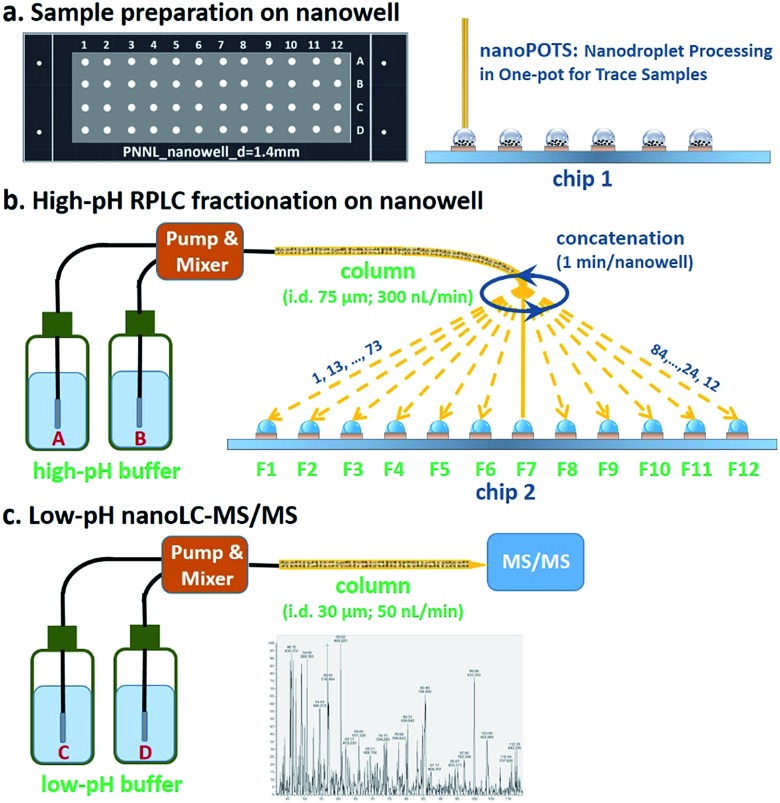
Workflow for nanowell-mediated 2D LC. (a) Nanogram biological samples or small populations of cells are prepared using nanoPOTS. (b) Samples are separated using high-pH nanoflow RPLC. The eluent from the high-pH RPLC separation is concatenated into nanowells and allowed to dry. (c) Samples in each nanowell are reconstituted and collected into capillaries for subsequent low-pH nanoLC-MS/MS analysis.

The workflow for the nanowell-mediated 2D LC is shown in Fig. 1. The preparation of the cellular material to produce ready-to-analyze peptides utilized our recently developed nanoPOTS method (Fig. 1a) on a microfluidic glass chip (chip 1).^24^ While not the focus of this study, this nanoPOTS platform provides efficient processing of small biological samples into ready-to-analyze peptides. The prepared samples were separated using an 84 min high-pH nanoLC separation performed at a flow rate of 300 nL min^–1^. The peptides eluted from the high-pH RPLC separation were concatenated at 1 minute intervals into 4 or 12 nanowells on another microfluidic glass chip (chip 2) using an in-house-developed robotic system (Fig. 1b and Video S1†) and allowed to dry. Each nanowell thus contained 7 or 21 pooled fractions, depending on the concatenation scheme employed (shown for 12 pooled fractions in Fig. 1b). Each fraction was reconstituted in 0.1% formic acid in water and collected into a capillary for the subsequent low-pH nanoLC-MS/MS analysis.

The nanowell platform effectively overcomes the limitations of traditional offline 2D LC platforms by reducing processing volumes to hundreds of nanoliters and total surface exposure to ∼1.5 mm^2^ to minimize surface losses. It also enables the use of a much narrower bore column (75 μm) and a low flow rate (300 nL min^–1^) for the first dimension RP fractionation, thus leading to smaller volumes and improved separation performance relative to conventional high-pH RPLC separations.^31^ In addition, it is noteworthy that the nanowell-mediated fractionation avoids the need for time-consuming vacuum-assisted evaporative concentration, as the nanoliter solvent volumes readily evaporate during the fractionation process.

### Sample recovery from nanowells

**Fig. 2:**
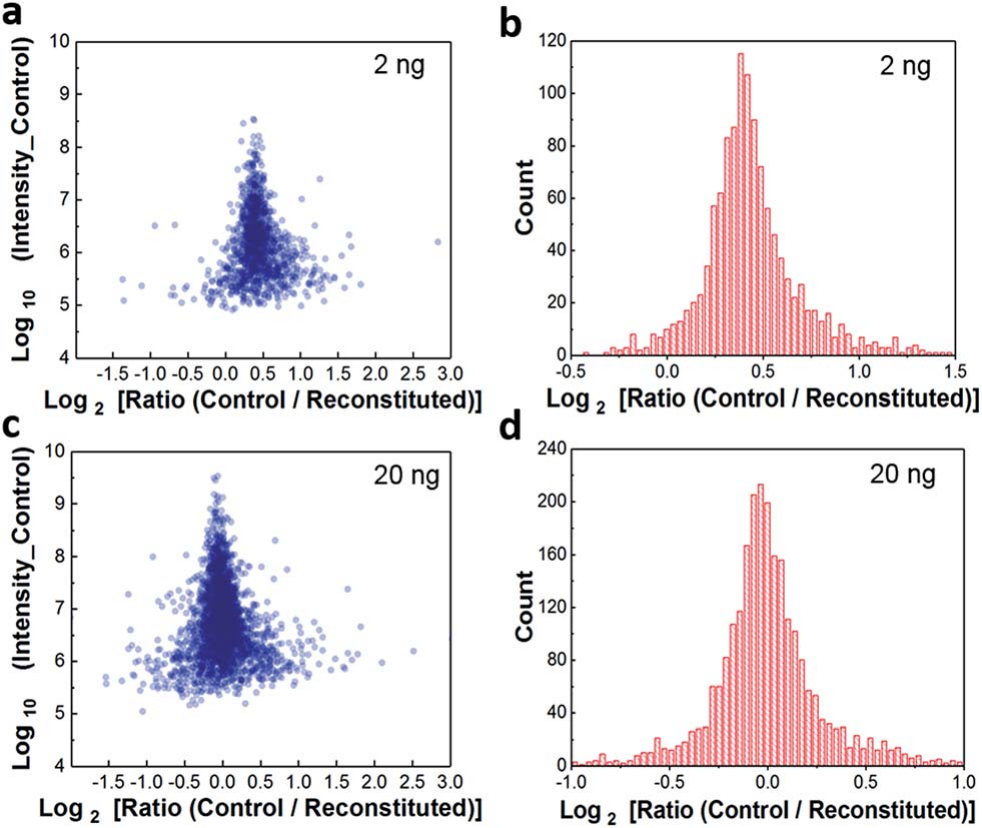
Sample recovery from nanowells following reconstitution of 2 ng (a, b) and 20 ng (c, d) HeLa digest. The log_2_ ratio of intensities between direct-loaded samples (control, *n* = 3) and reconstituted samples (reconstituted, *n* = 3) (a, c) and their histograms (b, d).

Because the workflow involves dispensing high-pH-RPLC-separated peptides into nanowells and allowing them to dry prior to reconstituting for low-pH RPLC analysis, it was important to characterize the extent to which peptides were lost to the nanowell surfaces. We evaluated sample recovery from the nanowells by dispensing two different amounts of unfractionated HeLa digest, 2 ng and 20 ng, into nanowells, evaporating the solvent, reconstituting in 0.1% formic acid, and analyzing by low-pH nanoLC-MS. These reconstituted samples were compared to control samples of the same starting amounts that were analyzed directly. We plotted protein intensity ratios *versus* the average intensity of control samples (Fig. 2a). The median log_2_-transformed ratio was 0.41 for 2 ng samples (Fig. 2b), indicating that the median recovery was 75% and that only ∼0.5 ng of peptides were lost to the nanowell surface. For all 1211 quantifiable proteins, 1162 proteins (96%) have recoveries >50%. Although ∼25% of the total peptide mass was lost to the nanowells, we observed a modest 15% reduction in peptide identifications and an 8% reduction in protein identifications (Fig. S2†). For the 20 ng samples, no significant difference in protein intensities or protein identifications was observed between control and reconstituted samples, indicating negligible peptide losses for the higher sample loadings (Fig. 2c and d, and S2†). Together, these results indicate minimal sample losses to the nanowell surfaces despite drying the samples in the nanowells, thus ensuring efficient sample transfer between the high and low pH separation modes.

### Characterization using 50 ng of HeLa digest

**Fig. 3:**
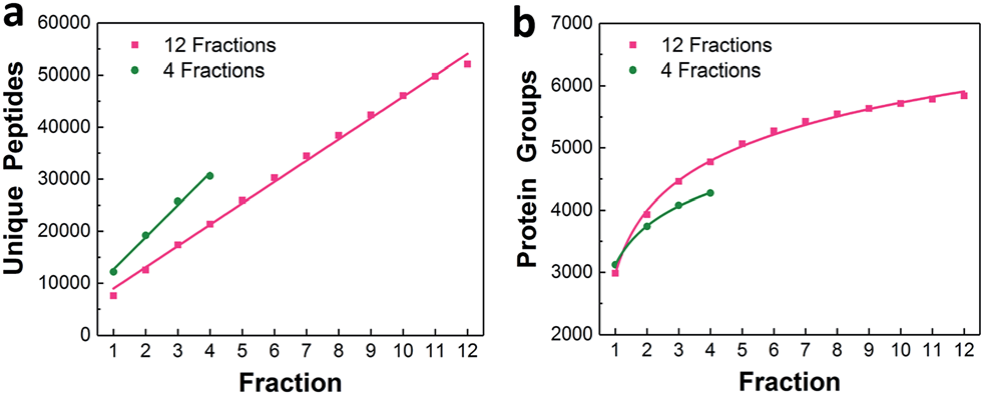
Proteome coverage. Cumulative identified unique peptides (a) and proteins (b) for 4 and 12 fractions using 50 ng of HeLa digest.

We characterized the performance of the nanowell-mediated 2D LC system for 4 and 12 fractions using 50 ng of HeLa digest as a model sample, which is at least 10 times smaller than any sample previously analyzed by 2D LC. The identified peptides and proteins were nearly uniformly distributed across the pooled fractions (Fig. S3†). This uniform distribution can be ascribed to the automated concatenation strategy, which combines fractions from across the high-pH separation space into each nanowell with a minimal overlap and a wide separation window, thus more effectively utilizing the second dimension separation.^13^,^32^,^33^ We also assessed the redundancy of identified unique peptides and the overlap between neighboring fractions for 4 and 12 fractions (Fig. S4†). The overlap of identified unique peptides was 23–25% for 4 fractions and 20–24% for 12 fractions. Cumulative peptide and protein identifications across the fractions are shown in Fig. 3a and b. Consistent with prior work,^14^ the identified peptides increased linearly with the number of fractions, while diminishing returns were observed for protein identifications.

**Fig. 4:**
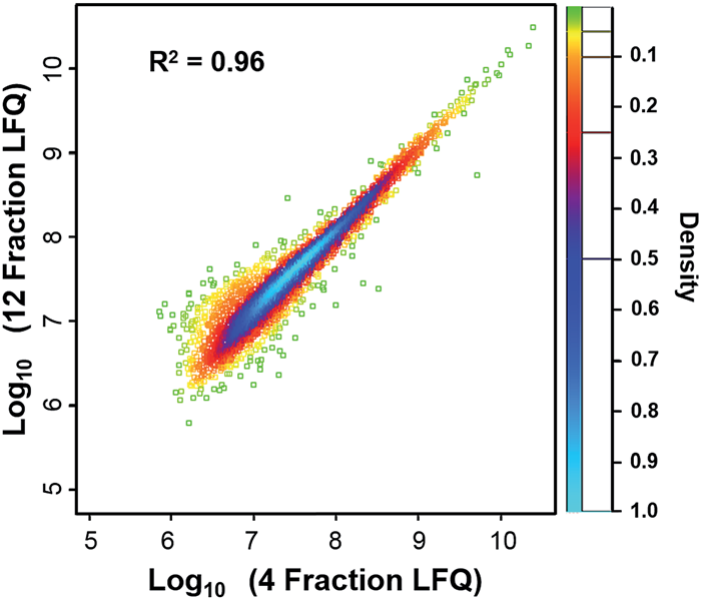
Scatter plot of the proteins quantified from 12 and 4 fractions by using 50 ng HeLa digest. The Pearson coefficient for the scatter plot was calculated to be 0.96, indicating that the proteins quantified from 12 and 4 fractions had a high correlation and were very similar at the quantitative level.

We compared the protein identifications among 1- (unfractionated using only the low-pH separation), 4- and 12-fraction samples using 50 ng of HeLa digest. As shown in Table 1, the identified unique peptides were 20 399, 30 605, and 52 069, corresponding to 2987, 4271, and 5839 proteins with the increase in fractions. Compared with the 1D separation, the 4-fraction approach increased the protein identifications by 43% and the 12-fraction approach provided a 95% increase. Based on the copy number estimates provided by Wiśniewski *et al.*,^34^ this doubling of proteome coverage requires the ability to detect ∼30-fold less abundant species. Clearly, more fractions can significantly increase the protein coverage, but at the expense of longer total analysis time. Thus, the extent of fractionation should take this trade-off into consideration. To the best of our knowledge, this is the lowest sample amount (*i.e.* 50 ng HeLa digest) reported for in-depth proteomics employing multidimensional separations, where far larger samples, in the microgram or milligram range, were typically used (see Table S1†).^32^,^33^,^35^ Moreover, label-free protein quantification between the 4-fraction and 12-fraction analyses indicated excellent reproducibility with a Pearson correlation coefficient of 0.96 (Fig. 4). These results demonstrate that the nanowell-mediated 2D LC approach enables high sensitivity, high coverage, and reproducible proteomic analyses of low-nanogram samples.

**Table 1:** Identified peptides and proteins for 12 and 4 fractions, as well as with a 1D separation using 50 ng of HeLa digest

	Peptides	Protein groups
12 fractions	52 069	5839
4 fractions	30 605	4271
1D separation	20 399	2987

### Proteome profiling of ∼650 HeLa cells

**Fig. 5:**
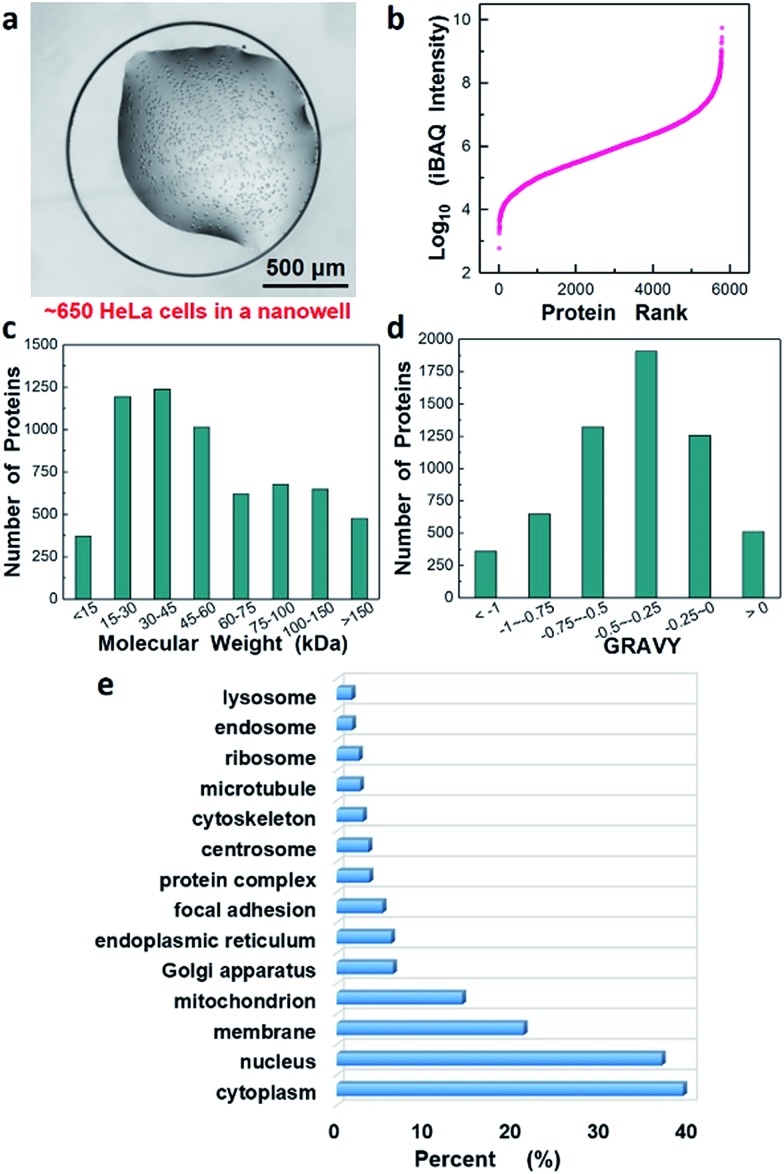
In-depth proteome analysis of ∼650 HeLa cells following concatenation into 12 fractions. (a) HeLa cells in a nanowell for sample preparation and analysis, (b–d) proteins ranked according to their (b) abundance levels (log_10_ iBAQ values), (c) molecular weight, and (d) GRAVY. (e) Gene Ontology annotations for Cellular Component^42^ of the identified proteins.

Next, we evaluated the performance of the nanowell-mediated 2D LC system for in-depth proteome profiling of a population of just ∼650 HeLa cells, which is equal to ∼100 ng of starting material (Fig. 5a). Sample preparation utilized our recently developed nanoPOTS approach,^24^ which enables the high-efficiency preparation of nanoscale protein samples by reducing processing volumes to the nanoliter range. However, the proteome coverage for HeLa cells was limited to ∼3000 proteins due to the 1D LC separation. Herein, by incorporating the nanowell-mediated 2D LC platform with 12 fractions, we were able to confidently identify 52 964 unique peptides, corresponding to 5805 proteins from the 650 cells. Analysis of unfractionated samples of similar size, as well as from 4 fractions, yielded similar coverage as the corresponding analyses of 50 ng of bulk-prepared HeLa digest (Fig. S5†). We have thus achieved a >10-fold reduction in required sample amounts to obtain a coverage of ∼6000 proteins (see Table S1†).^36^–^41^

We estimated the dynamic range of protein abundance, the distribution of molecular weights and the hydrophilicity for the 5805 identified proteins. Intensity-based absolute quantitation (iBAQ) was used for the estimation. Fig. 5b shows the proteins ranked according to their abundance level (log_10_-transformed iBAQ values). The dynamic range spanned 7 orders of magnitude from 6.0 × 10^2^ to 5.6 × 10^9^. Most identified proteins had a molecular weight (MW) below 100 kDa, and 1125 were found to have MW > 100 kDa (Fig. 5c). Interestingly, we also identified 511 highly hydrophobic proteins with GRAVY values >0 (Fig. 5d), which are generally associated with membrane proteins. Fig. 5e further revealed Gene Ontology annotations of Cellular Component (GOCC) for the total 5805 proteins and 1237 proteins were annotated as membrane proteins that perform a variety of vital functions.

### Application to single human islet cells

Finally, to demonstrate the potential application for deep proteome profiling of small populations of cells obtainable from clinical specimens, the present system was applied to profile human islets obtained by laser microdissection from a presymptomatic type 1 diabetic donor. Ten randomly selected islet thin sections (10 μm in thickness) were processed (Fig. S6†) using the nanoPOTS platform and the digest was analyzed by nanowell-mediated 2D LC. Based on previous studies,^24^,^43^ only <1 islet equivalents (IEQ) or ∼1000 cells were contained in the analysis. Using 12 fractions, we are able to identify 31 272 unique peptides and 6311 proteins (ESI 1†). Interestingly, a number of important low-abundance islet cell transcription factors^44^ such as PDX1, NKX 2.2, NKX 6.1, ARX, ISL1, and PAX6 were confidently identified. Together, these results demonstrated that the integration of a nanowell-mediated 2D LC system with the nanoPOTS platform could provide an unprecedented capability for deep proteome profiling of ultralow amounts of biological materials or small cell populations, including those from clinical specimens.

## Conclusions

In conclusion, we have developed a nanowell-mediated 2D LC approach that enables deep proteome profiling of low-nanogram samples corresponding to <1000 mammalian cells. This approach significantly reduces adsorptive losses to surfaces, allowing nanoscale samples to be fractionated into nanowells, dried and reconstituted for subsequent analysis. Analysis of ∼650 HeLa cells and ∼1000 human islet cells demonstrates the high sensitivity and high coverage for proteome profiling with nanoscale sample requirements. This nanowell-mediated 2D LC approach should enable comprehensive proteome characterization of various biomaterials, including clinical specimens with limited supplies such as fine needle aspiration biopsies, organoids, purified primary cells, circulating tumor cells and tissue substructures obtainable by laser microdissection.

## Conflicts of interest

There are no conflicts to declare.

## Supplementary Material

Supplementary information

Supplementary movie

Supplementary information
