# Supplementary material for: Nanowell-mediated two-dimensional liquid chromatography enables deep proteome profiling of <1000 mammalian cells
Source: Chem Sci. 2018 Jul 18;9(34):6944–51. doi: 10.1039/c8sc02680g (PMC6124911; doi:10.1039/c8sc02680g)
Supplement: Supplementary file 1 [file SC-009-C8SC02680G-s001.pdf]

Electronic Supplementary Material (ESI) for Chemical Science.
This journal is © The Royal Society of Chemistry 2018

Supporting Information

Nanowell-Mediated Two-Dimensional Liquid Chromatography Enables Deep Proteome Profiling of <1000 Mammalian Cells

Maowei Dou [†a], Ying Zhu [†a], Andrey Liyu [a], Yiran Liang [a], Jing Chen [b], Paul D. Piehowski [c], Kerui Xu [c], Rui Zhao [a], Ronald J. Moore [c], Mark A. Atkinson [b], Clayton E. Mathews [b], Wei-Jun Qian [c], and Ryan T. Kelly [*a]

[a.] *Environmental Molecular Sciences Laboratory, Pacific Northwest National Laboratory, Richland, WA 99354, USA.*

[b.] *Department of Pathology, Immunology and Laboratory Medicine, University of Florida, Gainesville, FL 32611, USA.*

[c.] *Biological Sciences Division, Pacific Northwest National Laboratory, Richland, WA 99354, USA.*

[†] Authors contributed equally to this work.

[*] Email: ryan.kelly@pnnl.gov

Figure S1

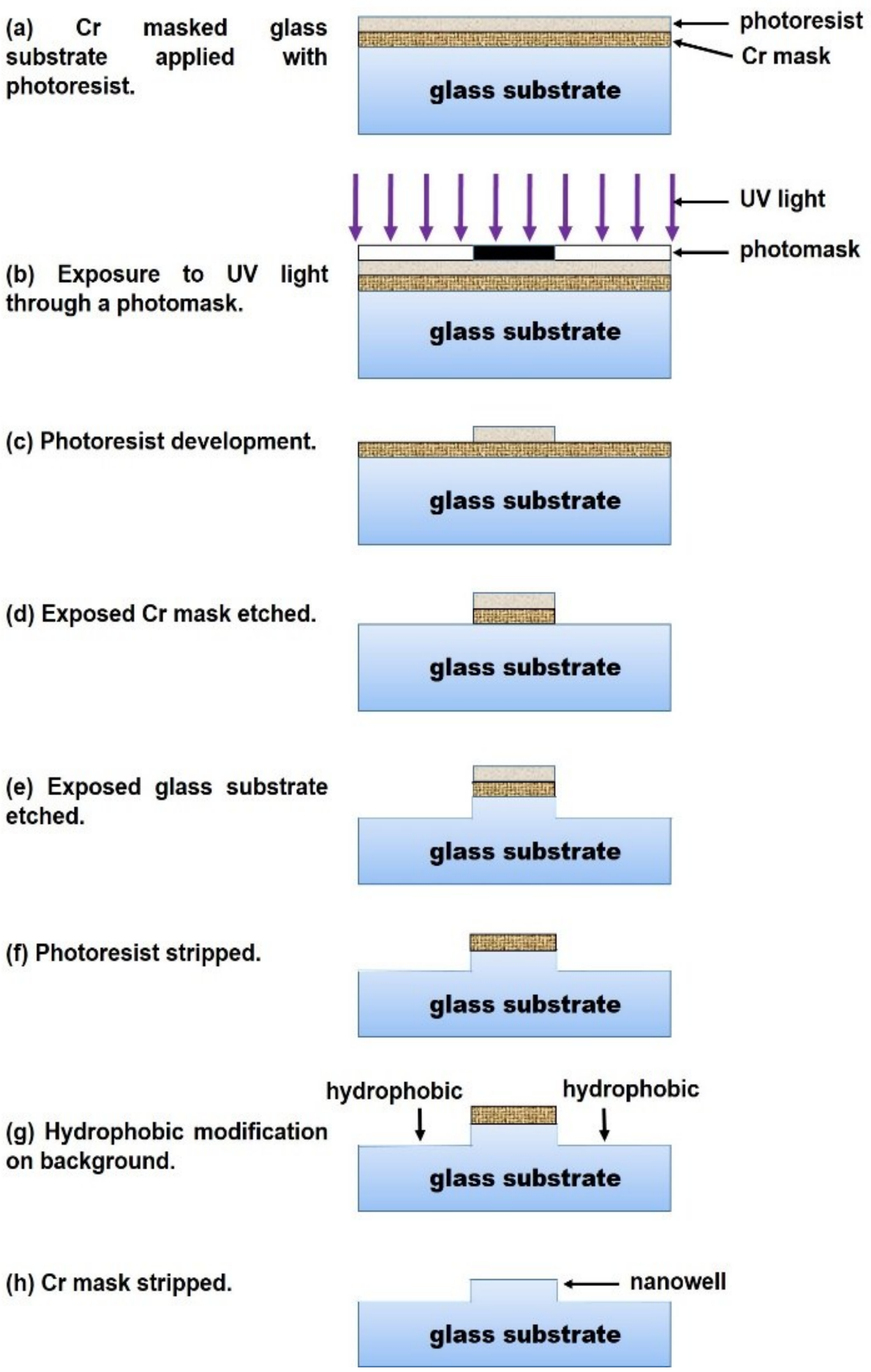

Fig. S1 Photolithography-based microfabrication process for nanowell chip on glass substrate.

Figure S2

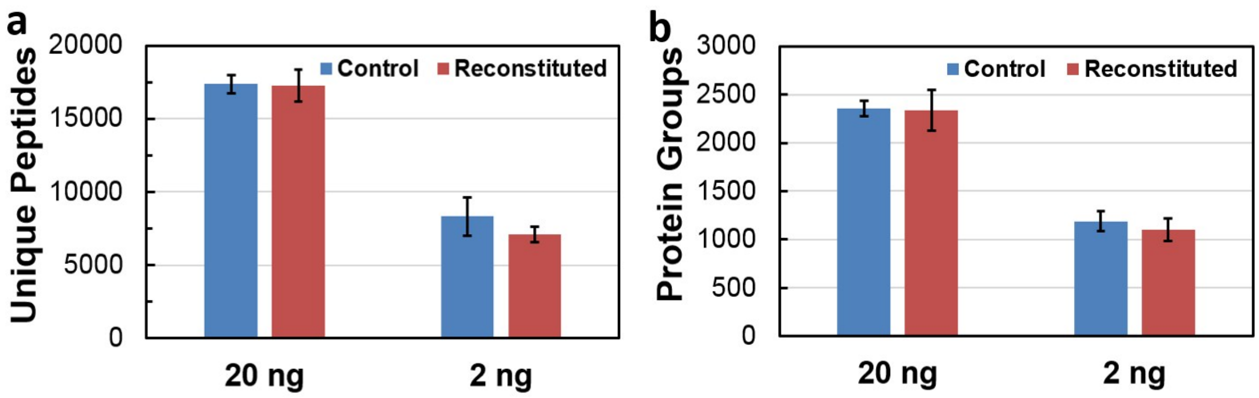

Fig. S2 Identifications of unique peptides (a) and proteins (b) from direct loading samples (control) and reconstituted samples (reconstituted) using 20 ng and 2 ng HeLa digest. The error bars represent the standard deviation.

Figure S3

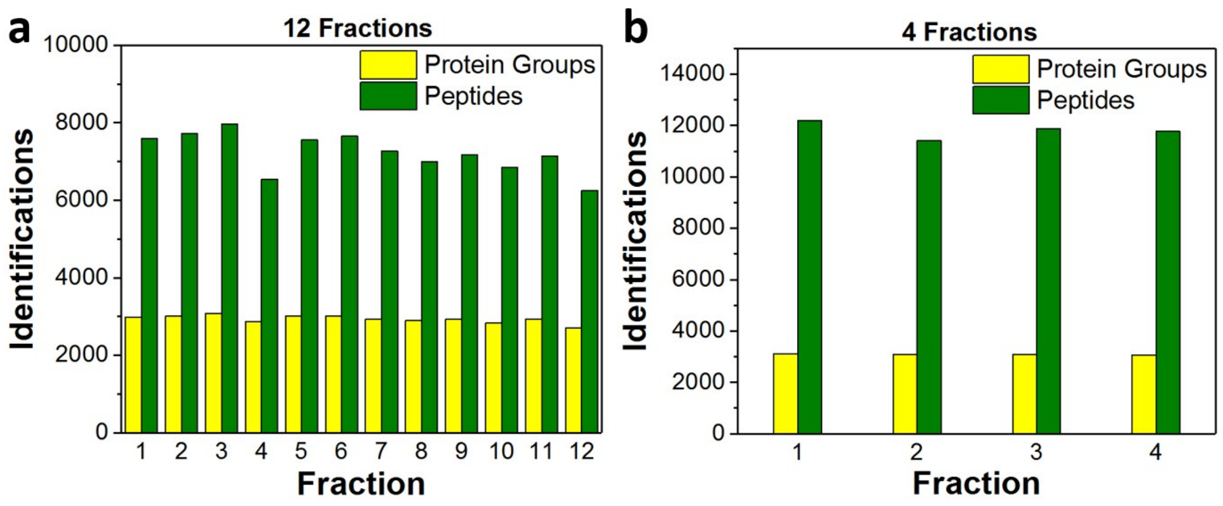

Fig. S3 Identifications of unique peptides and proteins per fraction from 50 ng HeLa digest following concatenation into 12 (a) and 4 (b) fractions. The identified proteins were uniformly distributed at each fraction, which were 2827-3209 with an average value of 3055 for 12 fractions, and were 3184-3243 with an average value of 3211 for 4 fractions.

Figure S4

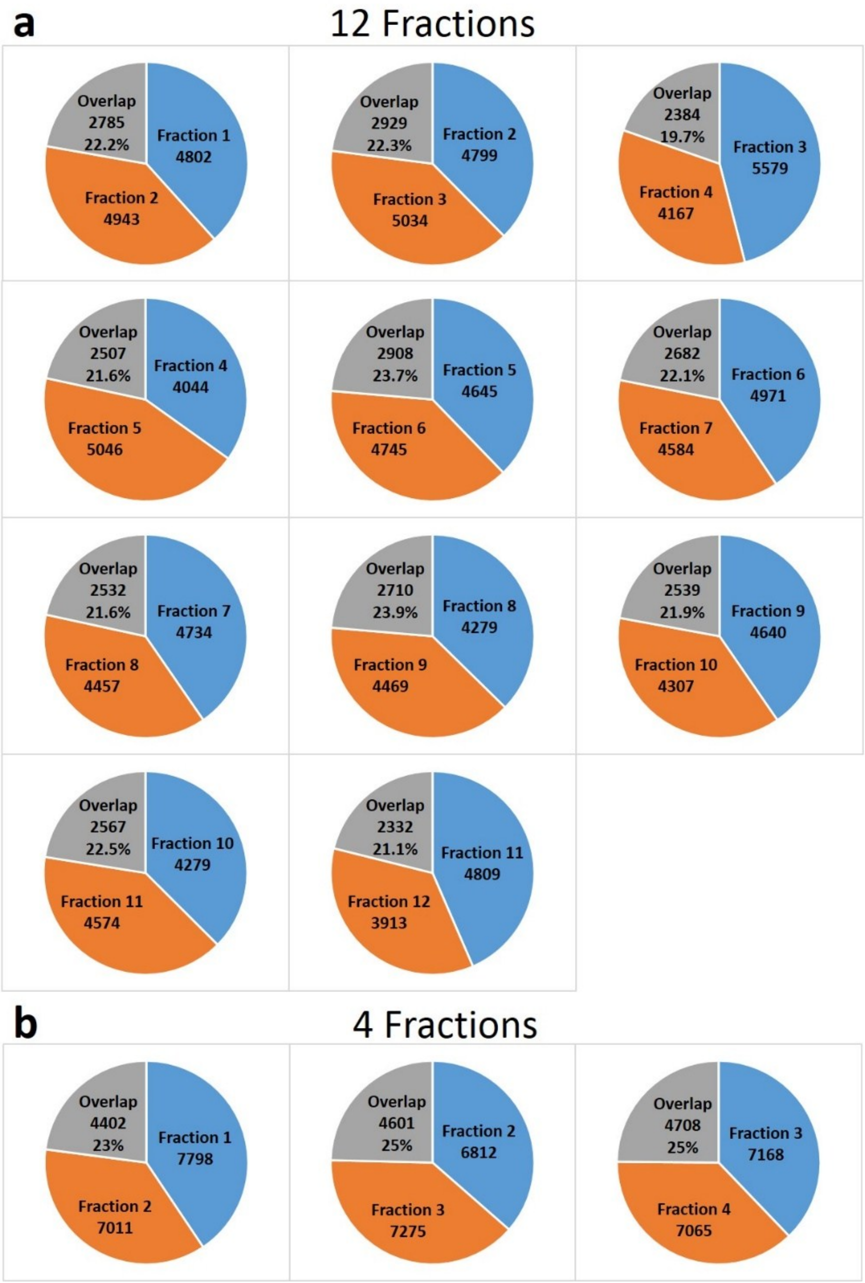

Fig. S4 Identifications of unique peptides and overlaps between neighboring fractions from 50 ng HeLa digest following concatenation into 12 (a) and 4 (b) fractions. The overlaps of identified unique peptides were 19.7%-23.9% for 12 fractions, and were 23%-25% for 4 fractions.

Table S1

Table S1. Previously reported protein identification results starting with microgram samples and thousands of cells

Sample Amount	Cell Line	Identified Protein Number	Separation Method	Reference
365 µg	Human plasma	800-1682	SCX-LC	1
300 µg	MCF10A	4363	2D LC	2
300 µg	MCF10A	2789	SCX-LC	2
150 µg	U2OS	8471	2D LC	3
150 µg	DMS	7969	2D LC	3
150 µg	HepG2	7323	2D LC	3
150 µg	MFM	7800	2D LC	3
150 µg	Yeast	4306	2D LC	3
150 µg	293T	8956	2D LC	3
15 µg	Arabidopsis chloroplast	685	2D LC	4
15 µg	Arabidopsis chloroplast	862	SCX-LC	4
0.8 µg (16-cell embryo)	Frog (*Xenopus laevis*) blastomeres	1466	LC	5
0.5 µg	HeLa	< 6000	2D LC	6
6600 cells	HeLa	< 6000	2D LC	6
5500 cells	Breast tumor cells	2556	LC	7
5000 cells	MCF-7	619	LC	8
3000 cells	Breast tumor cells	2282	LC	9
3000 cells	HeLa	2055	LC	10
2500 cells	CMF-7	491	LC	8
2000 cells	MCF-7	3370	LC	11
2000 cells	239T	1270	LC	12
1000 cells	MCF-7	237	LC	8
1000 cells	MCF-7	2512	LC	11
1000 cells	HeLa	1536	LC	10

Figure S5

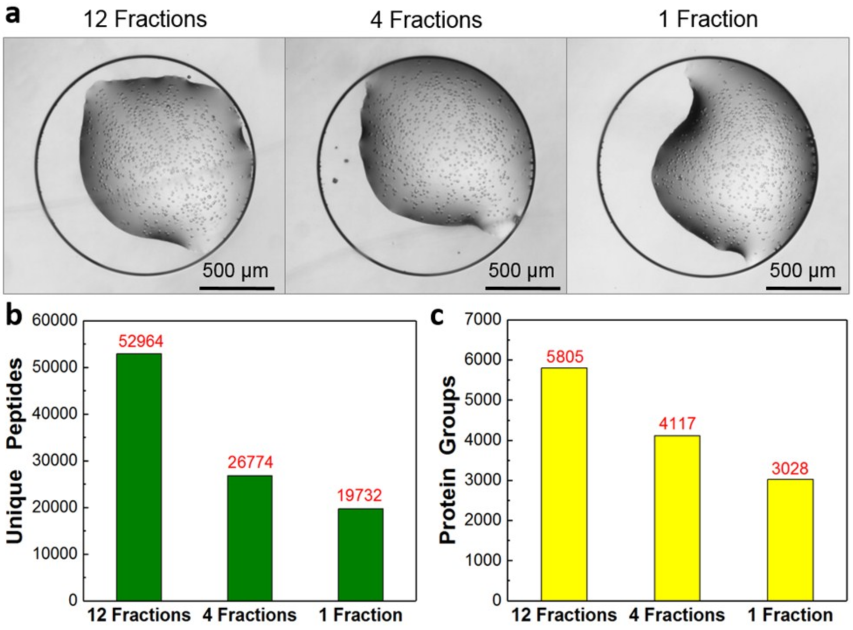

Fig. S5 In-depth proteome analysis of ~650 HeLa cells. (a) ~650 HeLa cells in a nanowell for sample preparation and analysis at 12 and 4 fractions, as well as 1 fraction (unfractionated), respectively; (b-c) Identified peptides (b) and proteins (c) at 12 and 4 fractions, as well as no fraction by using ~650 HeLa cells.

Figure S6

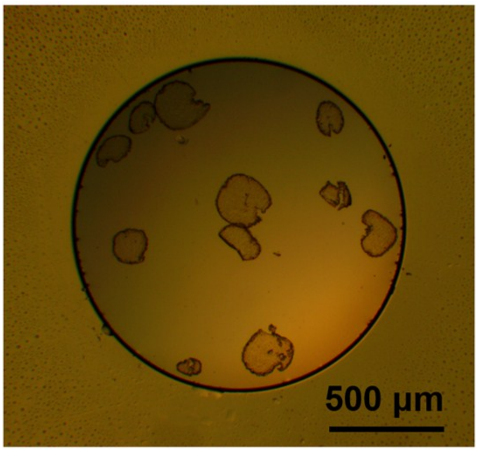

Fig. S6 Ten randomly selected islet thin sections in a nanowell for sample preparation and analysis.

S.3 References

1. Y. Shen, J. M. Jacobs, D. G. Camp, R. Fang, R. J. Moore, R. D. Smith, W. Xiao, R. W. Davis and R. G. Tompkins, *Analytical Chemistry*, 2004, **76**, 1134-1144.
2. Y. Wang, F. Yang, M. A. Gritsenko, Y. Wang, T. Clauss, T. Liu, Y. Shen, M. E. Monroe, D. Lopez-Ferrer, T. Reno, R. J. Moore, R. L. Klemke, D. G. Camp II and R. D. Smith, *Proteomics*, 2011, **11**, 2019-2026.
3. H. Wang, S. Sun, Y. Zhang, S. Chen, P. Liu and B. Liu, *Journal of Chromatography B*, 2015, **974**, 90-95.
4. R. P. W. Kong, S. O. Siu, S. S. M. Lee, C. Lo and I. K. Chu, *Journal of Chromatography A*, 2011, **1218**, 3681-3688.
5. L. Sun, K. M. Dubiak, E. H. Peuchen, Z. Zhang, G. Zhu, P. W. Huber and N. J. Dovichi, *Analytical Chemistry*, 2016, **88**, 6653-6657.
6. N. A. Kulak, P. E. Geyer and M. Mann, *Molecular & Cellular Proteomics*, 2017, **16**, 694-705.
7. A. Umar, H. Kang, A. M. Timmermans, M. P. Look, M. E. Meijer-van Gelder, M. A. den Bakker, N. Jaitly, J. W. M. Martens, T. M. Luider and J. A. Foekens, *Molecular & Cellular Proteomics*, 2009, **8**, 1278-1294.
8. N. Wang, M. Xu, P. Wang and L. Li, *Analytical Chemistry*, 2010, **82**, 2262-2271.
9. A. Umar, T. M. Luider, J. A. Foekens and L. Paša-Tolić, *Proteomics*, 2007, **7**, 323-329.
10. J. R. Wiśniewski, P. Ostasiewicz and M. Mann, *Journal of Proteome Research*, 2011, **10**, 3040-3049.
11. S. Li, B. D. Plouffe, A. M. Belov, S. Ray, X. Wang, S. K. Murthy, B. L. Karger and A. R. Ivanov, *Molecular & Cellular Proteomics*, 2015, **14**, 1672-1683.
12. W. Chen, S. Wang, S. Adhikari, Z. Deng, L. Wang, L. Chen, M. Ke, P. Yang and R. Tian, *Analytical Chemistry*, 2016, **88**, 4864-4871.
